# Treatment of Hepatitis B Virus Cirrhosis

**DOI:** 10.5812/hepatmon.6113

**Published:** 2012-05-30

**Authors:** Mario Rizzetto

**Affiliations:** 1 Department of Gastroenterology, Molinette, University of Torino, Torino, Italy

**Keywords:** Hepatitis B Virus, Liver Cirrhosis, Therapeutics

An analysis of cohorts of patients with compensated cirrhosis caused by the Hepatitis B Virus (HBV) indicates that the disease decompensates at an annual rate of 1.5% to 5%. Following decompensation, the 5-year survival rate has varied from 14% to 35% [1]; the 5-year survival rate in untreated patients is higher in patients decompensating from variceal bleeding than in those decompensating from ascites (29 vs. 16%) [2]. Given its ominous course, it is of no surprise that international guidelines recommend treating HBV cirrhosis to control HB viremia, prevent the dire complications of the disease, and increase survival. Interferon is an unﬁt treatment solution. It is contrain dicated in decompensated cirrhosis and of limited use in compensated cirrhosis [3][4]. HBV can usually only be controlled for temporarily; the cytokine cannot be given for long because of side effects and bacterial infection; and exacerbation of liver disease can decompensate the disease. Antiviral therapy for HBV cirrhosis entered common practice following the seminal study of Dr. Liaw [5], who showed that the treatment of compensated HBV cirrhosis with Lamivudine signiﬁcantly diminishes the risk of decompensation and complications; speciﬁcally, after 36 months of therapy, only 9% of the patients in Lamivudine treatment experienced progression of the disease, versus 21% of those in the placebo group, a difference so signiﬁcant that it prompted early termination of the trial. Unfortunately, Lamivudine treatment is aggravated by a high risk of viral resistance and hepatitis ﬂares; these conditions can cause rapid deterioration of the liver disease and death [6]. Adefovir, the next antiviral to be developed, conﬁrmed the efficacy of treatment for HBV cirrhosis, in particular in the transplantation setting. In two studies, patients with lamivudine-refractory-decompensated HBV were given Adefovir for 1 year; at the 1-year mark, these patients had signiﬁcantly improved the Meld score versus baseline [7][8]. However Adefovir was not perfect, as it had a potential for nephrotoxicity (a particular risk in cirrhotic patients) and suppressed HBV replication only slowly. Furthermore, the patients’ experience with Lamivudine suggested that therapy was of no immediate beneﬁt to the cirrhotic patients, some of whom died early despite therapy. A survival advantage became apparent only after 6 months of therapy, indicating that patients with the most severe disease progressions had not improved regardless of HBV repression. The message is obviously to treat cirrhosis with the efficacious drugs before the disease reaches a point of no return [9]. The advent of second-generation antivirals such as Entecavir and Tenofovir has offered better therapeutic prospects. Studies were completed on the efficacy and safety of Entecavir both in patients with compensated and decompensated HBV cirrhosis. In cases of compensated cirrhosis, Entecavir was shown to inhibit HBV replication more rapidly and effectively than either Lamivudine or Adefovir, with no signiﬁcant toxicity [10][11]. Entecavir is safe and effective in decompensated cases as well. In a cohort of 70 consecutive Korean patients with decompensated HBV cirrhosis [12], treatment led to more than a 2-point decrease of the Child-Pugh score for 50% of patients. Additionally, HBV-DNA became undetectable in almost 90% of patients, HBeAg loss was experienced by 48% of patients, and a 1-year transplant-free survival rate was experienced by 87% of patients. Controlling HBV was warranted in all patients during the 1-year follow-up. In a study in Taiwan [13], the outcome of decompensated HBV cirrhosis was compared between patients treated for 96 weeks with Entecavir and patients treated for 96 weeks with Adefovir. Entecavir was superior, reducing HBV-DNA at all-time points and improving the Meld score at Week 48 of therapy (down 2.6 points from baseline). In 2009 concern was raised that Entecavir might cause lactic acidosis because the drug was reported to induce this metabolic condition in patients with advanced liver disease [14]. However, further studies have dispelled this concern; only one case of lactic acidosis was reported among Entecavir recipients in clinical trials [15], and in a small controlled study of critically ill patients, no difference in lactacidemia was observed between patients who received Entecavir and those who went untreated [16]. Similarly good performance has been reported for Tenofovir [17]. One study compared 45 patients with decompensated cirrhosis who were treated with Tenofovir, 45 patients who were treated with Tenofovir and Emtricitabine, and 22 patients who were treated with Entecavir. At week 48, HBV-DNA was undetectable in 70.5% of the ﬁrst group, 87.8% of the second group, and 72.7% of the third group. Liver enzymes returned to normal in 57%, 76%, and 55% of patients, respectively and seroconversion to anti-HBe occurred in 21%, 13%, and 0%, respectively. Long-term control of HBV with antivirals has important beneﬁts. Survival distinctly increases in HBV cirrhosis given antivirals compared to untreated patients [2]. The impact has been dramatic in the liver transplant setting; the widespread application of antiviral therapy for HBV has contributed to diminish the demand for HBV transplants; a distinct proportion of patients exhibit clinical ameliorations that are signiﬁcant enough to permit their withdrawal from the transplant list [8]. Unexpected yet most encouraging, cumulative data from registrative trials of Entecavir and Tenofovir have shown that long-term control of HBV results in signiﬁcant reduction of ﬁbrosis. Of 57 patients responding for 3 years to Entecavir for whom paired liver biopsies were available, 88% exhibited a regression of ﬁbrosis with a decrease in the Ishak ﬁbrosis score of more than 1 point, including 10 patients with advanced ﬁbrosis [18]. Of 94 patients responding for 240 weeks to Tenofovir for whom paired biopsies were available, 72% exhibited a regression by at least 2 points in the Ishak ﬁbrosis score [19]. In keeping with the histological data, the hepatic venous pressure gradient diminished in all but 1 of 19 patients just after 1 year of Lamivudine therapy [5]; thus amelioration of liver ﬁbrosis at morphology appears to be functionally matched by a decrease of portal hypertension. The unfortunate news is that antiviral treatment diminishes but does not eliminate the risk of hepatocellular carcinoma. In a systematic analysis of 3,881 Lamivudine-treated and 534 untreated patients during a median 46-month period, hepatocellular carcinoma was diagnosed in 6.4% of untreated patients but also in 2.8% of treated patients [20]. The EASL Clinical Practice Guidelines [1] recommend treating HBV cirrhosis that exhibits any level of HBV-DNA, either compensated or decompensated, with potent antivirals that have very low risk of resistance (i.e. Tenofovir and Entecavir). Lamivudine is not recommended; if used because of local policy, Lamivudine should be used in combination with Tenofovir.

## References

[1] (2009). EASL Clinical Practice Guidelines: management of chronic hepatitis B. J Hepatol.

[2] Das K, Datta S, Pal S, Hembram JR, Dhali GK, Santra A (2010). Course of disease and survival after onset of decompensation in hepatitis B virus-related cirrhosis. Liver Int.

[3] Perrillo R, Tamburro C, Regenstein F, Balart L, Bodenheimer H, Silva M (1995). Low-dose, titratable interferon alfa in decompensated liver disease caused by chronic infection with hepatitis B virus. Gastroenterology.

[4] Hoofnagle JH, Di Bisceglie AM, Waggoner JG, Park Y (1993). Interferon alfa for patients with clinically apparent cirrhosis due to chronic hepatitis B. Gastroenterology.

[5] Liaw YF, Sung JJ, Chow WC, Farrell G, Lee CZ, Yuen H (2004). Lamivudine for patients with chronic hepatitis B and advanced liver disease. N Engl J Med.

[6] Di Marco V, Marzano A, Lampertico P, Andreone P, Santantonio T, Almasio PL (2004). Clinical outcome of HBeAg-negative chronic hepatitis B in relation to virological response to lamivudine. Hepatology.

[7] Schiff ER, Lai CL, Hadziyannis S, Neuhaus P, Terrault N, Colombo M (2003). Adefovir dipivoxil therapy for lamivudine-resistant hepatitis B in pre- and post-liver transplantation patients. Hepatology.

[8] Schiff E, Lai CL, Hadziyannis S, Neuhaus P, Terrault N, Colombo M (2007). Adefovir dipivoxil for wait-listed and post-liver transplan tation patients with lamivudine-resistant hepatitis B: final longterm results. Liver Transpl.

[9] Fontana RJ, Hann HW, Perrillo RP, Vierling JM, Wright T, Rakela J (2002). Determinants of early mortality in patients with decompensated chronic hepatitis B treated with antiviral therapy. Gastroenterology.

[10] Schiff E, Simsek H, Lee WM, Chao YC, Sette H, Jr., Janssen HL (2008). Efficacy and safety of entecavir in patients with chronic hepatitis B and advanced hepatic fibrosis or cirrhosis. Am J Gastroenterol.

[11] Leung N, Peng CY, Hann HW, Sollano J, Lao-Tan J, Hsu CW (2009). Early hepatitis B virus DNA reduction in hepatitis B e antigenpositive patients with chronic hepatitis B: A randomized international study of entecavir versus adefovir. Hepatology.

[12] Shim JH, Lee HC, Kim KM, Lim YS, Chung YH, Lee YS (2010). Efficacy of entecavir in treatment-naive patients with hepatitis B virusrelated decompensated cirrhosis. J Hepatol.

[13] Liaw YF, Raptopoulou-Gigi M, Cheinquer H, Sarin SK, Tanwandee T, Leung N (2011). Efficacy and safety of entecavir versus adefovir in chronic hepatitis B patients with hepatic decompensation: a randomized, open-label study. Hepatology.

[14] Lange CM, Bojunga J, Hofmann WP, Wunder K, Mihm U, Zeuzem S (2009). Severe lactic acidosis during treatment of chronic hepatitis B with entecavir in patients with impaired liver function. Hepatology.

[15] Keating GM (2011). Entecavir: a review of its use in the treatment of chronic hepatitis B in patients with decompensated liver disease. Drugs.

[16] Marzano A, Marengo A, Marietti M, Rizzetto M (2011). Lactic acidosis during Entecavir treatment in decompensated hepatitis B virusrelated cirrhosis. Dig Liver Dis.

[17] Liaw YF, Sheen IS, Lee CM, Akarca US, Papatheodoridis GV, Suet- Hing Wong F (2011). Tenofovir disoproxil fumarate (TDF), emtricitabine/ TDF, and entecavir in patients with decompensated chronic hepatitis B liver disease. Hepatology.

[18] Chang TT, Liaw YF, Wu SS, Schiff E, Han KH, Lai CL (2010). Long-term entecavir therapy results in the reversal of fibrosis/cirrhosis and continued histological improvement in patients with chronic hepatitis B. Hepatology.

[19] Marcellin P, Buti M, Gane EJ, Krastev Z, Flisiak R, Germanidis G (2011). Five years of treatment with TenofovirDF (TDF) for Chronic Hepatitis B (CHB) infection is associated with sustained viral suppression and significant regression of histological fibrosis and cirrhosis. AASLD.

[20] Papatheodoridis GV, Lampertico P, Manolakopoulos S, Lok A (2010). Incidence of hepatocellular carcinoma in chronic hepatitis B patients receiving nucleos(t)ide therapy: a systematic review. J Hepatol.

